# A Prototypical Template for Rapid Face Detection Is Embedded in the Monkey Superior Colliculus

**DOI:** 10.3389/fnsys.2020.00005

**Published:** 2020-02-06

**Authors:** Quang Van Le, Quan Van Le, Hiroshi Nishimaru, Jumpei Matsumoto, Yusaku Takamura, Etsuro Hori, Rafael S. Maior, Carlos Tomaz, Taketoshi Ono, Hisao Nishijo

**Affiliations:** ^1^System Emotional Science, Faculty of Medicine, University of Toyama, Toyama, Japan; ^2^Primate Center and Laboratory of Neurosciences and Behavior, Department of Physiological Sciences, Institute of Biology, University of Brasília, Brasilia, Brazil; ^3^Laboratory of Neuroscience and Behavior, CEUMA University, São Luis, Brazil

**Keywords:** innate recognition, face detection, face features, superior colliculus, monkey

## Abstract

Human babies respond preferentially to faces or face-like images. It has been proposed that an innate and rapid face detection system is present at birth before the cortical visual pathway is developed in many species, including primates. However, in primates, the visual area responsible for this process is yet to be unraveled. We hypothesized that the superior colliculus (SC) that receives direct and indirect retinal visual inputs may serve as an innate rapid face-detection system in primates. To test this hypothesis, we examined the responsiveness of monkey SC neurons to first-order information of faces required for face detection (basic spatial layout of facial features including eyes, nose, and mouth), by analyzing neuronal responses to line drawing images of: (1) face-like patterns with contours and properly placed facial features; (2) non-face patterns including face contours only; and (3) nonface random patterns with contours and randomly placed face features. Here, we show that SC neurons respond stronger and faster to upright and inverted face-like patterns compared to the responses to nonface patterns, regardless of contrast polarity and contour shapes. Furthermore, SC neurons with central receptive fields (RFs) were more selective to face-like patterns. In addition, the population activity of SC neurons with central RFs can discriminate face-like patterns from nonface patterns as early as 50 ms after the stimulus onset. Our results provide strong neurophysiological evidence for the involvement of the primate SC in face detection and suggest the existence of a broadly tuned template for face detection in the subcortical visual pathway.

## Introduction

Newborn babies orient toward faces and schematic face-like figures (three filled circles on a bright ellipse; Johnson et al., 1991). During this early period in life when the cortical visual systems are still immature, the subcortical visual areas including the superior colliculus (SC) are proposed to convey facial information to the extrastriate cortices (Johnson, 2005). This proposal is further supported by studies in other species. For example, infant monkeys reared without exposure to faces still reacted stronger to conspecific pictures as well as human faces compared with non-face objects (Sackett, 1966; Sugita, 2008). In other vertebrate species, newly hatched chicks or chicks reared in dark, in which the optic tectum is the homolog of the mammal SC (Butler and Hodos, 2005), show preference for the similar schematic face-like figures and photos of human faces (Rosa-Salva et al., 2010, 2011). These behavioral data suggest that the vertebrate brain may have an innate face processing system or an innate prototypical face template (“Conspec” Morton and Johnson, 1991).

The SC, one of the subcortical visual areas, is phylogenetically old and might support innate visual recognition in vertebrates (Sewards and Sewards, 2002; Carr, 2015). Accumulating evidence from human neuropsychological studies suggests the existence of the subcortical face processing pathway, which consists of the SC, pulvinar, and amygdala (Tamietto and de Gelder, 2010; Rafal et al., 2015). A blindsight patient with visual cortical lesions showed residual visual functions; they were able to unconsciously distinguish between normal faces and faces with arbitrarily placed facial features (Solcà et al., 2015), suggesting a subcortical involvement in visual processing. Furthermore, patients with those lesions could discriminate known from unknown faces, gaze directions, and facial expressions (de Gelder et al., 1999; Tamietto and de Gelder, 2010; Burra et al., 2013; Solcà et al., 2015; Bertini et al., 2019). Human functional magnetic resonance imaging (fMRI) and monkey neurophysiological studies suggest that the subcortical pathway conveys coarse information of visual stimuli including faces (Morris et al., 2001; Vuilleumier et al., 2003; Chen et al., 2018; Burra et al., 2019). These neuropsychological results suggest that the subcortical visual pathway, which receives both direct and indirect visual inputs from the retina, functions as an alternative pathway that bypasses V1 and directly reaches the extrastriate cortex (Weiskrantz, 1996; Berman and Wurtz, 2010, 2011; Pessoa and Adolphs, 2010; Tamietto and de Gelder, 2010).

Face information processing consists of several different steps (Maurer et al., 2002; Tsao and Livingstone, 2008); (1) detection of faces based on first-order information (i.e., arrangement of facial features such as eyes, nose, mouth, etc.); (2) holistic processing of facial features (whole-face representation based on integration of facial features); and (3) facial discrimination of different individuals based on second-order information (discrimination of variance across faces). Although computational studies suggest that the first face detection step precedes the others and is important to make the later processes efficient, neural mechanisms of this step remain poorly understood (Tsao and Livingstone, 2008). Previous neurophysiological studies indicated that facial photos activated monkey SC and pulvinar neurons and their latencies were shorter than those in the striate cortex (Nguyen et al., 2013, 2014). These studies strongly suggest that the subcortical visual pathway is involved in face detection. However, it is also possible that these neurons could respond to individual facial features regardless of their relative positions within the facial contours. Thus, whether SC neurons respond to first-order information of faces (basic layout of facial features including eyes, nose, and mouth) itself or not remains unknown. To test this possibility, we analyzed the responses of monkey SC neurons to face-like and non-face patterns, in which facial features were either orderly or randomly positioned within contours. Furthermore, the responses to inverted (upside down) and contrast-reversed patterns of the same face-like and non-face patterns were examined as well. We looked for neurons coding a face template in the SC which would respond stronger to both normal and contrast-reversed face-like patterns as well as upright and inverted face-like patterns than non-face patterns.

## Materials and Methods

### Subjects

In this study, two adults (one female and one male, 7.0 and 8.8 kg) monkeys (*Macaca fuscata*) were used. The rearing environment of animals was the same as that in previous studies (Le et al., 2013, 2014; Nguyen et al., 2013, 2014; Dinh et al., 2018). Individual monkeys were reared in a cage with food available *ad libitum*. The day before the experimental sessions, the monkey was subject to water restrictions in the home cage, and received juice rewards during the experimental sessions. They received additional water and vegetables after the training and recording sessions. The monkeys were handled according to the United States Public Health Service Policy on Human Care and Use of Laboratory Animals, the NIH Guide for the Care and Use of Laboratory Animals, and the Guidelines for Laboratory Animals in the University of Toyama. This study has been approved by the Committee for Animal Experiments and Ethics at the University of Toyama.

### Experimental Set Up

The experimental apparatus was the same as that used in our previous studies (Le et al., 2013, 2014; Nguyen et al., 2013, 2014; Dinh et al., 2018). Briefly, a chair on which the monkeys sat was placed 68 cm away from a 19-inch monitor for a behavioral task in a shielded room. The chair had a response button, and the monkey responded to a task by pressing the button (see below). Monkey’s eye-movements were observed by an infrared charge-coupled device (CCD) with a 33 ms time resolution during recording sessions (Matsuda, 1996). The monkey could obtain the juice reward through a small spout attached to the chair, which was controlled by an electromagnetic valve. Visual stimuli were generated by a stimulus generator (ViSaGe MKII Visual Stimulus Generator, Cambridge Research Systems, UK), which also controlled the timing of visual stimulus onset as well as the electromagnetic valve.

### Visual Stimuli

**Figure 1:**
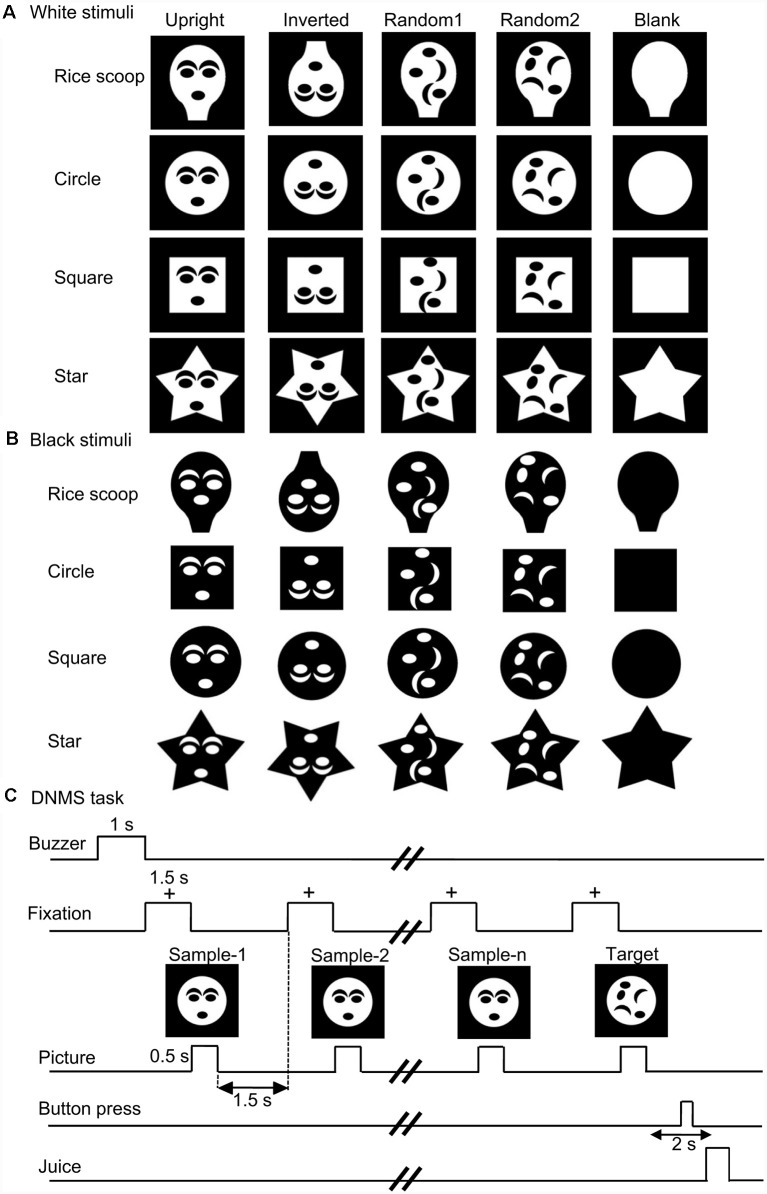
The visual stimulus set used in the present study **(A,B)** and task sequence **(C)**. **(A,B)** Twenty stimuli of four different contours (rice scoop, circles, square, star) in the white **(A)** and black **(B)** stimulus sets. The stimulus set for each contour consisted of five stimuli with the following different arrangement of facial features: (1) upright face-like pattern (upright); (2) inverted face-like pattern (inverted); (3) contour with randomly positioned facial features (random1); (4) contour with randomly positioned facial features (random2); and (5) contour without facial features (blank). **(C)** Stimulus sequence in the delayed non-matching-to-sample (DNMS) task, in which stimuli were sequentially presented with a delay between them.

Figures 1A,B show the visual stimuli used in this study. These stimuli consisted of two different sets of stimuli (white and black stimulus sets in Figures 1A,B, respectively). Each stimulus set included four stimulus groups with four different contours (rice scoop, star, circle, square). Each stimulus group included five visual stimuli (forms) that had the same contour; two face-like patterns (upright, inverted), and three non-face patterns [two random patterns (random1, random2) and blank contour only (blanks)]. Each face-like pattern consisted of one of four face contours and five facial features (two eyes, two eyebrows, one mouth), while the random patterns consisting of the same facial contour and features as in the face-like patterns, but the facial features were randomly positioned within the facial contour. The stimuli were grayscale images with their resolution of 170 × 170 pixels. All white visual stimuli were presented on the monitor with a black background of 0.7 cd/m^2^, whereas black stimuli were likewise presented on the monitor with a 45.27 cd/m^2^ white background. The luminance of each stimulus was measured by using a luminance meter (BM-7A; Topcon, Tokyo). Sizes of all stimuli in each stimulus set were adjusted so that stimuli had similar luminance. Luminance of the all stimuli in the white and black sets was 18.1–18.5 and 26.3–26.4 cd/m^2^, respectively. These stimuli were presented on the monitor (640 × 480 pixels), and the size of the stimuli was 3–4 × 3–4°.

### Behavioral Tasks

The task was the same as that used in our previous studies (Le et al., 2013, 2014; Nguyen et al., 2013, 2014; Dinh et al., 2018). Briefly, the monkeys were trained to discriminate the visual stimuli in a delayed non-matching-to-sample task (DNMS; Figure 1C). After a buzzer tone, the monkeys were required to fixate on a fixation cross within 0.5–1.0° window for 1.5 s. Then, a sample stimulus appeared for 500 ms (sample phase). With a 1.5-s interval, the same stimulus was presented again for 500 ms from 1 to 4 times. Finally, a different stimulus (a target stimulus) appeared for 1.5 s (target phase), and the monkey pressed the button in the chair within 2 s after the offset of the target stimulus to obtain a juice reward (0.8 ml). When the monkey made an error response, the trial was terminated with a 620-Hz buzzer tone. The intertrial intervals (ITI) was set to 15–25 s.

Thus, the monkey discriminated sample and target stimuli in each trial of the DNMS task. Stimulus pairs included stimuli in the same group of each stimulus set.

### Training and Surgery

The procedures were the same as those in our previous studies (Le et al., 2013, 2014; Nguyen et al., 2013, 2014; Dinh et al., 2018). Briefly, the monkeys were trained in the DNMS task. When performance levels of the monkeys reached a 96% correct rate, the monkeys received a surgery. In the surgery under anesthesia with medetomidine hydrochloride (0.5 mg/kg, i.m.) and ketamine hydrochloride (5 mg/kg, i.m.), a U-shaped plate made of epoxy resin was attached to the skull with the U-shaped plate being anchored to titanium bolts inserted into the skull with dental acrylic (Nishijo et al., 1988a,b; Tazumi et al., 2010). A small pin was also implanted into the skull as a reference, coordinates of which were calibrated in reference to the zero coordinates in the stereotaxic brain atlas of *Macaca fuscata* monkeys (Kusama and Mabuchi, 1970). Antibiotics were administered after the surgery. In the monkey retraining that started 10 days after the surgery, the monkeys performed the DNMS task with the head being painlessly fixed to the stereotaxic apparatus. The performance criterion (>90%) was again attained within 2 weeks.

### Electrophysiological Procedures and Data Acquisition

The procedures were the same as those used in our previous studies (Le et al., 2013, 2014; Nguyen et al., 2013, 2014; Dinh et al., 2018). Briefly, SC neuronal activity with a signal-to-noise ratio greater than 3:1 was recorded from a glass-insulated tungsten microelectrode (0.5–1.5 MΩ at 1 kHz), which was stereotaxically inserted into the SC. A multichannel acquisition processor (Plexon Inc., Dallas, TX, USA) system processed analog signals of SC neuronal activities, trigger signals of the events (onset of the visual stimuli, delivery of juice rewards, and button pressing), and the X-Y coordinates of eye position. These data digitized at 40-kHz were stored in a hard disk.

The digitized data were transferred to the NeuroExplorer program (Nex Technologies, Littleton, CO, USA) for spike sorting with cluster analysis (Off-line sorter, Plexon Inc., Dallas, TX, USA). All SC neurons were further analyzed by autocorrelograms: we confirmed that the refractory periods were greater than 2 ms in all SC neurons.

### Assessment of Visual Receptive Fields and Stimulus Presentation

Because SC neurons have retinotopically-organized receptive fields (RFs), we first checked responsive areas for SC neurons on the monitor. For this purpose, when monkeys fixated continuously at the fixation point, small white squares (1.0 × 1.0°) were presented for 500 ms at different locations on an 8 × 6 grid with 2.5 cm (2.0°) spacing (Supplementary Figure S1A in Supplementary Materials). The squares were pseudo-randomly presented at least three times at each grid location. Then, average peri-stimulus histograms in response to the white squares were computed for each grid location during the experiment. Significant (excitatory or inhibitory) responses were determined by comparing the neuronal activity between the 100-ms pre and the 500-ms post periods [Wilcoxon signed-rank (WSR) test (*P* < 0.05)]. Response areas (grids) were defined as the areas with a significant difference in WSR test (*P* < 0.05). In each grid location, response magnitude was computed according to the following definition; (the mean firing rate in the 500-ms post period) minus (the mean firing rate during the 100-ms pre-period). For each SC neuron, each largest response area, where the largest response magnitude was elicited, was determined. RFs of SC neurons were defined based on locations of the largest response areas in the visual field (VF) and divided into three groups: neurons with the largest response areas in the upper VF (SC neurons with upper RFs), neurons with the largest response areas in the lower VF (those with lower RFs), and neurons with the largest response areas in the central grid (those with central RFs). In the subsequent experiment with the DNMS task, the visual stimuli were presented on the monitor with their centers at the largest response area in each neuron.

### Analysis of SC Neuronal Responses

The procedures were the same as those used in our previous studies (Le et al., 2013, 2014; Nguyen et al., 2013, 2014; Dinh et al., 2018). Briefly, only SC neuronal responses to the sample stimuli, but not those to the target stimuli, were analyzed: firing rates during 100 ms before (pre) and 500 ms after (post) stimulus onset of the sample stimuli were estimated. The baseline firing-rate was estimated as follows; the mean firing rate during the 100-ms pre-period. Significant (excitatory or inhibitory) responses to each stimulus were determined by comparing neuronal activity between the 100-ms pre and the 500-ms post periods (WSR test, *P* < 0.05). The response magnitude was computed according to the following definition; (the mean firing rate in the 500-ms post period) minus (the mean firing rate in the 100-ms pre-period). In each neuron, the response magnitudes to the visual stimuli for the white stimulus set and those for the black stimulus set were separately analyzed by two-way ANOVA with “stimulus form” (arrangement of facial features) and “shape of the contours” as factors (*P* < 0.05) with Bonferroni *post hoc* tests (*P* < 0.05). Mean response magnitudes of population of SC neurons for the white stimulus set and those for the black stimulus set were also separately analyzed by repeated-measures two-way ANOVA with “stimulus form” and “shape of the contours” as factors (*P* < 0.05) with Bonferroni *post hoc* tests (*P* < 0.05). Linear relationships of response magnitudes between the white and black stimulus sets were analyzed by simple regression analysis (*P* < 0.05). Comparison of response ratios (response magnitudes to face-like patterns vs. those to non-face patterns) among neurons with different RFs was analyzed by two-way ANOVA with Bonferroni *post hoc* tests (*P* < 0.05).

Neuronal response latency was estimated by measuring the interval from stimulus onset to the time when the neuronal firing rate exceeded the mean ± 2.0 SD of the baseline firing rate. Thus, individual latencies to individual stimuli were estimated in each neuron. Mean response latencies of population of SC neurons for the white stimulus set and those for the black stimulus set were separately analyzed by repeated-measures two-way ANOVA with “stimulus form” and “shape of the contours” as factors (*P* < 0.05) with Bonferroni *post hoc* tests (*P* < 0.05). Linear relationships of response latencies between the white and black stimulus sets were analyzed by simple regression analysis (*P* < 0.05). Furthermore, in each neuron, one peri-event histogram for the whole white stimuli tested was constructed using the entire set of data for all trials and all-white stimuli tested, and that for the whole black stimuli tested was constructed. Then, latency for the whole white stimuli tested and that for the whole black stimuli tested were similarly estimated in each neuron. Comparison of response latencies to the white and black stimuli among neurons with different RFs or in different SC layers were analyzed by two-way ANOVA with Bonferroni *post hoc* tests (*P* < 0.05). All data were shown as mean ± SEM.

A previous study reported that microsaccades affected SC neuronal activity (Hafed and Krauzlis, 2010). Example traces of eye positions are shown in Supplementary Figure S2 in Supplementary Materials. As shown in these examples, microsaccades were observed in a few trials around 200–300 ms after stimulus onset, consistent with previous studies (Hafed et al., 2011; Tian et al., 2016). A recent study reported that microsaccades usually occur after the presentation of cues associated with large, but not small, reward in monkeys (Joshua et al., 2015). This suggests that microsaccades occur less frequently in response to visual stimuli associated with no reward, consistent with the present study in which only responses to the sample stimuli associated with no reward were analyzed in the DNMS task. Since microsaccades affect SC neuronal activity during 70 ms before and after onset of microsaccades (Hafed and Krauzlis, 2010) and microsaccades occurred around 200–300 ms after stimulus onset (Hafed et al., 2011; Tian et al., 2016), we also analyzed response magnitudes during the initial 100 ms after stimulus onset, during which microsaccades are supposed to do not affect SC neuronal activity.

### Multidimensional Scaling (MDS) Analysis

To analyze representation of the stimulus by population activity of the SC neurons, activity of the SC neurons with central RFs (*n* = 32) was analyzed by MDS (Le et al., 2013, 2014; Nguyen et al., 2013, 2014, 2016; Dinh et al., 2018). MDS constructs a stimulus space in which relationships among stimuli are shown (Young, 1987). Briefly, response magnitudes of the 32 SC neurons to the 40 visual stimuli during first (epoch 1) and second (epoch 2) 50-ms post periods were analyzed by the MDS: in each epoch, data matrices of neural activity (40 stimuli × 32 visually responsive neurons) were analyzed by MDS. The MDS program computed stimulus distances between all possible pair of the stimuli, and the program with PROXSCAL procedure (SPSS statistical package, ver. 16) placed each visual stimulus in stimulus spaces (Euclidean distance in this study) based on the stimulus distances (Shepard, 1962; Kruskal, 1964).

The clusters of the visual stimuli in the MDS spaces were analyzed using the two- and multiple-group discriminant analyses (SPSS statistical package, ver. 16). Significance of group separation in the discriminant analyses was assessed by Wilks’ Lambda test (*p* < 0.05).

#### Stereotaxic Localization and Histology

After the recording, several small electric lesions (20–30 μA for 30 s) were stereotaxically made within and around the SC under anesthesia (Nguyen et al., 2014). Then, the monkeys were deeply anesthetized (sodium pentobarbital, 100 mg/kg, i.m.), and perfused with 0.9% saline as well as 10% buffered formalin. The monkey brains, removed from the skulls, were cut into 50-μm sections, which were stained with cresyl violet. The locations of the visually responsive neurons were plotted on the actual tissue sections based on the coordinates of the recording sites and those of the lesions. The locations of the SC neurons in the two monkeys were plotted together on the SC sections of one monkey.

## Results

### Coherent Responses to White and Black Face-Like Patterns

**Figure 2:**
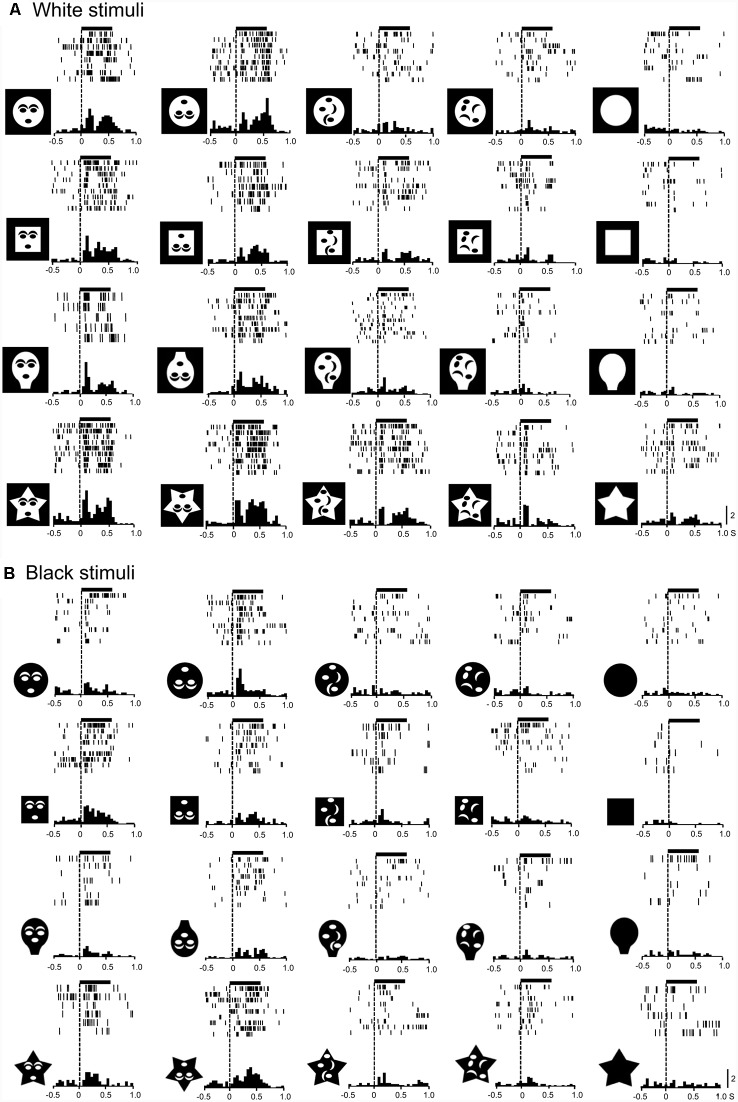
Visual responses of an superior colliculus (SC) neuron to the white **(A)** and black **(B)** stimulus sets. Horizontal bars above the raster displays indicate the stimulus presentation period (500 ms). The vertical dotted line in each of the raster displays and histograms indicate the stimulus-onset point. Calibration at the right bottom of the figure: number of spikes per trial in each bin. Bin width, 50 ms. Zero on the abscissa indicates the onset of the stimuli.

Monkey SC neurons were recorded, while the monkeys performed the DNMS task. Of 158 visually responsive SC neurons, 146 neurons were tested with all visual stimuli. Based on the response areas with the largest response magnitudes in the VF, 146 neurons were divided into three groups: neurons with the largest response areas in the upper VF (SC neurons with upper RFs, *n* = 46), neurons with lower RFs (*n* = 68), and those with central RFs (*n* = 32; for definition of RF, see “Materials and Methods” section). An example of an SC neuron with a central RF, where the largest responses were elicited by stimuli at the center of the display, is shown in Supplementary Figure S1 (Supplementary Materials). Figure 2 shows responses of the same SC neuron to the white and black stimulus sets. This neuron responded strongly to the upright and inverted face-like patterns with the four contours in both the white (A) and black (B) stimulus sets. Statistical analyses of the response magnitudes indicated that this SC neuron responded stronger to the upright and inverted face-like patterns than the other stimuli in the white set of the stimuli (Supplementary Figure S3A in Supplementary Materials) as well as in the black stimulus set (Supplementary Figure S3B in Supplementary Materials). Furthermore, response magnitudes to the white and black stimuli of the same forms (20 white stimuli vs. 20 black stimuli) were significantly correlated (Supplementary Figure S3C in Supplementary Materials).

**Figure 3:**
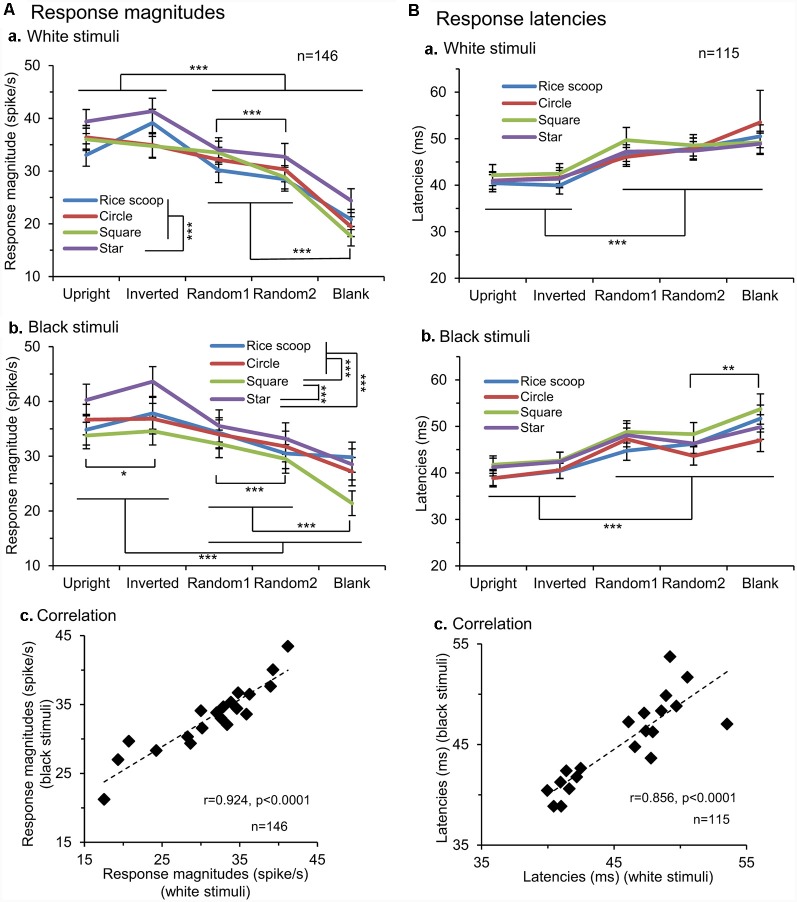
Comparison of the response magnitudes **(A)** and latencies **(B)** of the SC neurons. **(A)** Comparison of the response magnitudes to the white (a) and black (b) stimulus sets among the five visual stimuli, and correlation of the response magnitudes between the white and black stimulus sets (c). The significant difference by Bonferroni tests after repeated measures two-way ANOVA at *P < 0.05 and ***P < 0.001, respectively. **(B)** Comparison of the response latencies to the white (a) and black (b) stimulus sets among the five visual stimuli, and correlation of the response latencies between the white and black stimulus sets (c). The significant difference by Bonferroni tests after repeated measures two-way ANOVA at *P < 0.05, **P < 0.01 and ***P < 0.001, respectively.

We applied the same analysis to a total of 146 responsive neurons tested with all stimuli (Figure 3A). The mean response magnitudes to the face-like patterns were significantly larger than those to the non-face patterns in both white (Aa) and black (Ab) stimulus sets (Bonferroni tests after repeated measures two-way ANOVA, *P* < 0.001). Second, mean response magnitudes to the nonface random patterns (random1 and random2) were significantly larger than those to the blanks in both white (Aa) and black (Ab) stimulus sets (Bonferroni tests after repeated measures two-way ANOVA, *P* < 0.001). Third, simple linear regression analysis indicated that the mean response magnitudes to the white stimulus set were significantly and positively correlated with those to the black stimulus set (*F*_(1,18)_ = 105.749, *P* < 0.0001; *r* = 0.924; Ac). Thus, the SC neurons responded stronger to the face-like patterns regardless of contrast polarity, suggesting that activity of the SC neurons was dependent at least on the stimulus form itself.

Response latencies of SC neurons ranged widely from 10 to 350 ms. Consistent with the previous study using similar visual stimuli (Nguyen et al., 2014), there were two peaks of response latencies: those with short-latencies (10–100 ms) and those with long-latencies (>100 ms). The mean latency of the neurons with short-latencies was 36.0 ± 1.3 ms (*n* = 143), while that of the neurons with long-latencies was 183.9 ± 26.9 ms (*n* = 15). We further analyzed response latencies to the individual visual stimuli; response latencies to the all individual stimuli were estimated in 115 SC neurons with short-latencies (Figure 3B). The mean response latencies of the SC neurons to the face-like patterns (upright and inverted) were significantly shorter than those to the other non-face patterns in both the white (Ba) and black (Bb) stimulus sets (Bonferroni tests after repeated measures two-way ANOVA, *P* < 0.001). Furthermore, simple linear regression analysis revealed that the mean response latencies to the white stimulus set were significantly and positively correlated with those to the black stimulus set (*F*_(1,18)_ = 49.271, *P* < 0.0001; *r* = 0.856; Bc). The results again indicated that the response characteristics of the SC neurons were dependent on the stimulus form.

### Coherent Responses to Face-Like Patterns Across Different SC Layers and Different RFs

Similar coherent responses to the white and black face-like patterns were replicated when the data in different SC layers or different RFs were separately analyzed [see “Supplementary Results” (Supplementary Figures S4–S9) in Supplementary Materials].

### Effects of RFs on Coherency to White and Black Stimuli and Selectivity to Face-Like Patterns

**Figure 4:**
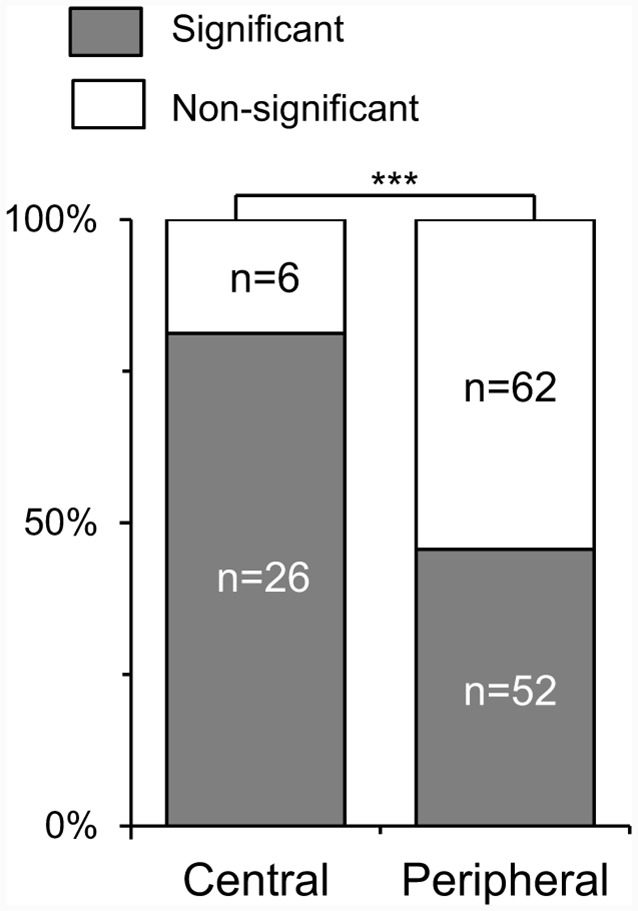
Ratios of the SC neurons with a significant correlation of response magnitudes between the white and black stimulus sets. Central, SC neurons with the central receptive fields (RFs); peripheral, SC neurons with the peripheral RFs; the significant difference at ***P < 0.001.

Although SC neurons responded similarly to the white and black stimuli as a whole, there was a significant difference in coherency among SC neurons with different RFs. Figure 4 shows comparison of ratios of the SC neurons with a significant correlation between response magnitudes to the white and black stimulus sets. Of the responsive 146 SC neurons tested with the all stimuli (SC neurons with the central RFs, *n* = 32; those with the peripheral RFs, *n* = 114), 78 neurons exhibited significant correlations (SC neurons with central RFs, *n* = 26; those with peripheral RFs, *n* = 52). Ratios of SC neurons with significant correlation were significantly greater in the SC neurons with the central RF than SC neurons with the peripheral RFs (*x*^2^ test, *P* = 0.0004). These results suggest that the SC neurons with the central RFs were more selective to stimulus forms rather than contrast polarity.

**Figure 5:**
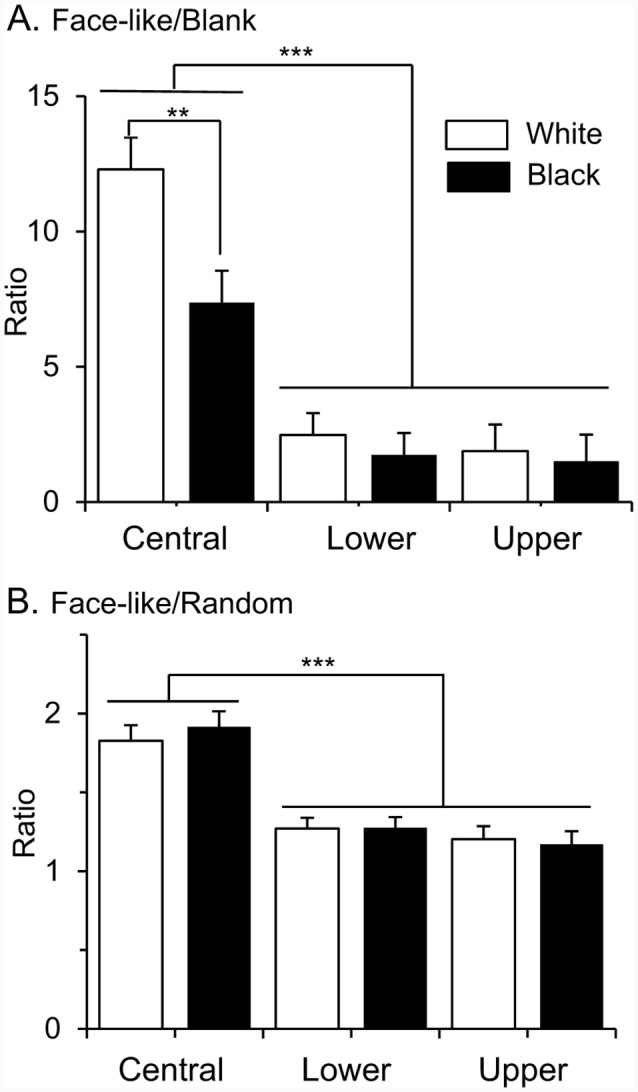
Ratios of response magnitudes to the face-like patterns to those to the blanks **(A)**, and ratios of response magnitudes to the face-like patterns to those to the random patterns **(B)**. Upper, lower, and central, SC neurons with the upper, lower, and central RFs, respectively. White, white stimulus set; black, black stimulus sets. The significant difference by Bonferroni tests after two-way ANOVA at **P < 0.01 and ***P < 0.001, respectively.

Furthermore, locations of the RFs affected selectivity to face-like patterns. To compare selectivity to face-like patterns, ratios of response magnitudes to the face-like patterns to those of non-face patterns were analyzed (Figure 5). When ratios of the response magnitudes to the upright and inverted face-like patterns to those to the blanks were compared by two-way ANOVA with “contrast polarity” and “RF” as factors, there was a significant main effect of RF (A; *F*_(2,286)_ = 35.2, *P* < 0.0001). *Post hoc* tests revealed that ratios of the response magnitudes to the face-like patterns to those to the blanks were significantly larger in the SC neurons with the central RFs than those with the lower and upper RFs (Bonferroni test, *P* < 0.0001). Furthermore, there was a significant interaction between contrast polarity and RF (*F*_(2,143)_ = 3.091, *P* = 0.049). *Post hoc* tests indicated that ratios of the response magnitudes to the face-like patterns to those to the blanks were significantly larger in the white stimulus set than the black stimulus set in the SC neurons with the central RFs (Bonferroni test, *P* = 0.003). Furthermore, when ratios of the response magnitudes to the face-like patterns to those to the non-face random patterns were compared in the same way, there was a significant main effect of RF (*F*_(2,286)_ = 32.582, *P* < 0.0001). *Post hoc* tests revealed that ratios of the response magnitudes to the face-like patterns to those to the non-face random patterns were significantly larger in the SC neurons with the central RFs than those with the lower and upper RFs (B; Bonferroni test, *P* < 0.0001).

**Figure 6:**
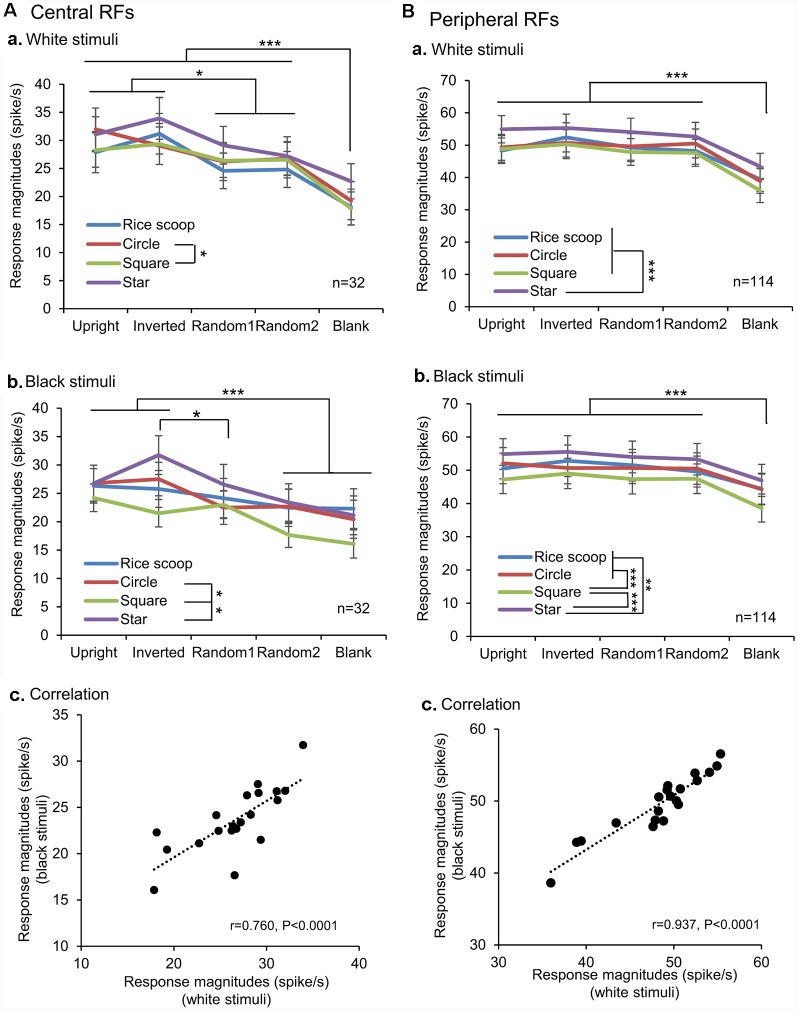
Comparison of the response magnitudes of the SC neurons with the central RFs **(A)** and those with the peripheral RFs **(B)** among the five visual stimuli. **(A,B)** Comparison of the response magnitudes to the white (a) and black (b) stimulus sets among the five visual stimuli, and correlation of the response magnitudes between the white and black stimulus sets (c). The significant difference by Bonferroni tests after repeated measures two-way ANOVA at *P < 0.05, **P < 0.01, and ***P < 0.001, respectively.

We also analyzed the response magnitudes to the visual stimuli during the initial 100 ms after stimulus onset in the SC neurons with the central and peripheral RFs (Figure 6). In the SC neurons with the central RFs (A), the mean response magnitudes to the face-like patterns in the white stimulus set were significantly larger than those to all nonface patterns (Bonferroni tests after repeated measures two-way ANOVA, *P* < 0.05; Aa). In the black stimulus set (Ab), the mean response magnitude to the inverted face-like pattern was significantly larger than those to the all nonface patterns (Bonferroni tests after repeated measures two-way ANOVA, *P* < 0.05), while the mean response magnitude to the upright face-like pattern was significantly larger than those to the nonface patterns including the random2 and blank (Bonferroni tests after repeated measures two-way ANOVA, *P* < 0.001). Simple linear regression analysis indicated that the mean response magnitudes to the white stimulus set were significantly and positively correlated with those to the black stimulus set (*F*_(1,18)_ = 24.544, *P* < 0.0001; *r* = 0.760; Ac). In the SC neurons with the peripheral RFs (B), the mean response magnitudes to the non-blank patterns (upright and inverted face-like patterns, ramdom1, and radom2) were significantly larger than those to the blank patterns in the white (Ba) and black (Bb) stimulus sets (Bonferroni tests after repeated measures two-way ANOVA, *P* < 0.001). Simple linear regression analysis indicated that the mean response magnitudes to the white stimulus set were significantly and positively correlated with those to the black stimulus set (*F*_(1,18)_ = 128.633, *P* < 0.0001; *r* = 0.937; Bc). Thus, the SC neurons with the central RFs were more sensitive to the face-like patterns than the SC neurons with peripheral RFs in the early latencies before 100 ms after stimulus onset.

### Representation of Face-Like Patterns

**Figure 7:**
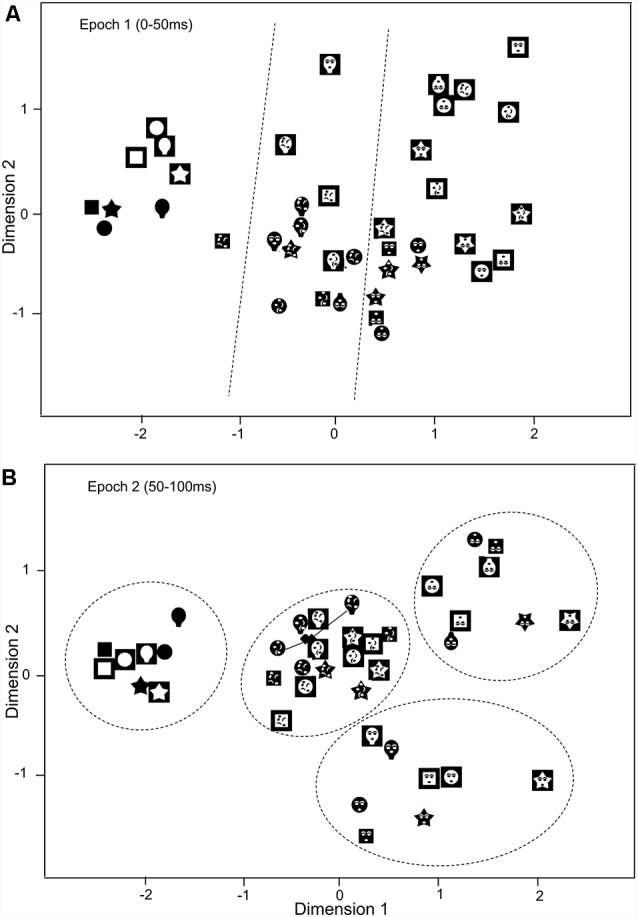
Distributions of the 40 visual stimuli in the two-dimensional space resulting from multidimensional scaling (MDS) of responses of SC neurons with central RF in epoch 1 **(A)** and epoch 2 **(B)**. In epoch 1 **(A)**, three groups of the visual stimuli (blanks, non-face random patterns, and face-like patterns) were separated (multiple-group discriminant analysis, *P* < 0.0001). Dotted lines indicate separation by multiple-group discriminant analysis. In epoch 2 **(B)**, the four groups were separated: blanks, non-face random patterns, inverted face-like patterns, and upright face-like patterns (multiple-group discriminant analysis, *P* < 0.0001). Two filled diamonds indicate true positions of the stimuli connected by solid lines.

The above results suggest that the SC neurons with central RFs were more selective to the face-like patterns. To analyze population coding of visual stimuli, the data sets of response magnitudes of the 32 visually responsive SC neurons with the central RFs in epochs 1 (0–50 ms), and 2 (50–100 ms) were analyzed MDS (Figure 7). The *r*^2^ and stress values for up to four dimensions indicated that two-dimensional spaces revealed the best results. In the two-dimensional space, *r*^2^ values of epochs 1 and 2 were 0.869 and 0.932, respectively. In epoch 1 (A), two groups were recognized: the blanks and the remaining stimuli (non-blanks). Two-group discriminant analyses revealed that the blanks were significantly separated from the non-blanks (*P* < 0.0001; Table 1). Furthermore, the face-like patterns were significantly separated from the non-face patterns (*P* = 0.003; Table 1). These findings suggest three groups of visual stimuli; blanks, non-face random patterns, and face-like patterns. The multiple-group discriminant analysis confirmed these three clusters of the visual stimuli (*P* < 0.0001). In epoch 2, group separation became clearer (B). Two-group discriminant analyses revealed that the blanks were significantly separated from the non-blanks (*P* < 0.0001; Table 1), and that the face-like patterns were significantly separated from the non-face patterns (*P* < 0.0001; Table 1). Furthermore, the upright face-like and inverted face-like patterns were separated from the remaining stimuli, respectively (both, *P* < 0.0001; Table 1). These findings suggest four clusters of the visual stimuli; blanks, non-face random patterns, inverted face-like patterns, and upright face-like patterns. The multiple-group discriminant analysis confirmed the four groups of the visual stimuli (*P* < 0.0001).

**Table 1:** Separation among the groups by two- and multiple-group discriminant analyses.

Groups	Stress value	*r*^2^	Correct ratio	*p*-value
Epoch 1	0.17717	0.86874		
Blank vs. non-blank			94.9%	<0.0001
Face-like vs. nonface			72.5%	<0.003
Face-like vs. random vs. blank			75.0%	<0.0001
Epoch 2	0.13386	0.93246		
Blank vs. non-blank			100%	<0.0001
Face-like vs. nonface			92.5%	<0.0001
Upright face-like vs. the remaining			100%	<0.0001
Inverted face-like vs. the remaining			97.5%	<0.0001
Face-like vs. random vs. blank			100%	<0.0001
Upright face-like vs. inverted face-like vs. random vs. blank			100%	<0.0001

*Two-dimensional coordinates of the 40 visual stimuli in multidimensional scaling (MDS) were used for discriminant analysis. The first column indicates stimulus groups that were tested by the discriminant analyses. Correct ratio, correct ratio of separation between the given groups; blank, blank stimuli (contour only); non-blank, visual stimuli except the blank stimuli; upright face-like, upright face-like patterns; inverted face-like, inverted face-like patterns; face-like, face-like patterns; nonface, nonface patterns; random, composite stimuli consisting of one of four contours and facial features in which facial features were randomly located (random1, random2); *p*-value, *p*-values in Wilks’ Lambda test; r^2^, r^2^ value in the MDS analysis*.

### Locations of SC Neurons

**Figure 8:**
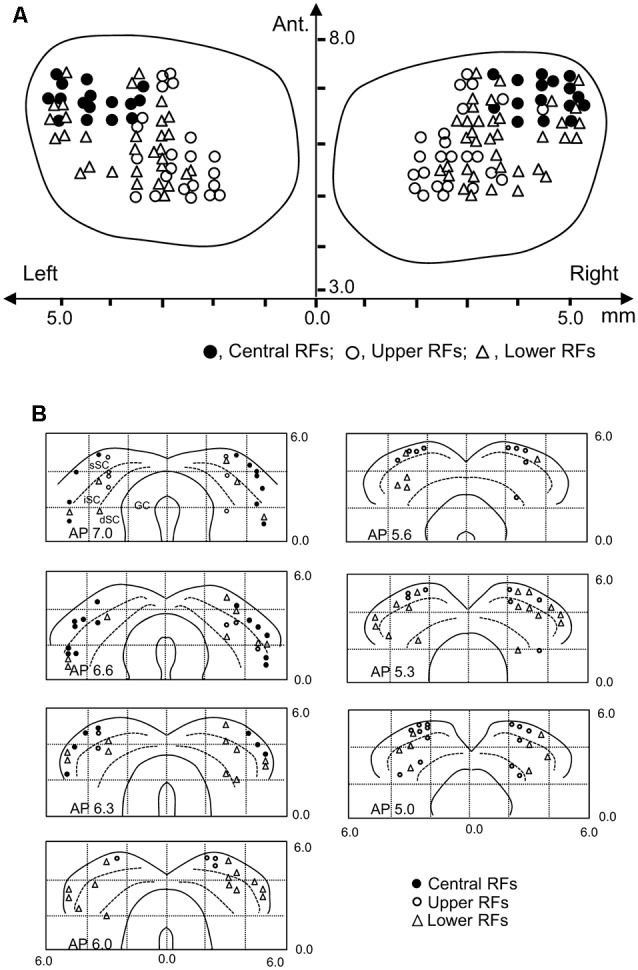
Recording sites of the SC neurons. **(A)** Distribution of the SC neurons in the top view of the SC. Ant., anterior (mm) from the line connecting the two external auditory canals. **(B)** Distribution of the depth of recording sites. Filled circles, SC neurons with the central RF; open circles, SC neurons with the upper RF; open triangles, SC neurons with the lower RF. sSC, superficial layers of the SC; iSC, intermediate layers of the SC; dSC, deep layers of the SC; GC, substantia grisea centralis.

Locations of the SC neurons (*n* = 146) tested with the all stimuli are shown in the top view of the SC (Figure 8A). The SC neurons with central RFs (filled circles) were located in the anterolateral part of the SC. The SC neurons with upper RFs (open circles) were located in the medial part of the recording sites, while the SC neurons with lower RFs (open blue triangles) were located in the lateral sites of the SC. Figure 8B shows locations of the SC neurons in each section of the SC in each A-P level.

## Discussion

### Neural Circuits for Detection of Face-Like Patterns

The present study indicated that the SC neurons were more sensitive to the face-like patterns than the nonface patterns. It is reported that SC neurons have different sizes of RFs from 2 to 20° with the deeper neurons having larger RFs (Goldberg and Wurtz, 1972; Li and Basso, 2008; Chen et al., 2019). Therefore, when the visual stimuli (3–4 × 3–4° in the present study) are presented in a response area with the largest responses, the stimuli would stimulate inside or a part of an RF depending on the shape and size of the RF of a given SC neuron. Thus, when RFs of SC neurons are smaller than the face-like patterns, those SC neurons may not receive whole information of the face-like patterns. However, it is possible that SC neurons with small RFs might receive visual information outside the RFs *via* other SC neurons. Previous studies reported intrinsic connections within the SC by horizontal interneurons as well as horizontal (lateral) collaterals of axons from SC neurons, which might allow integration of visual information from a large part of the VF (Behan and Appell, 1992; Meredith and Ramoa, 1998; Tardif et al., 2005). Consistently, a neurophysiological study reported both lateral excitation and inhibition in the mouse SC (Phongphanphanee et al., 2014). These lateral intrinsic connections within the SC might contribute to the relative selectivity of the SC neurons to the face-like patterns. A previous behavioral study also reported that deactivation of the superficial layer of the cat SC delayed learning of global, but not local, features of composite figures (Lomber, 2002), suggesting an SC involvement in integration of global visual information. Furthermore, the complex interaction between a stimulus and a RF could affect neuronal responses to the visual stimulus in a single neuronal level and might result in different patterns of neuronal responses to visual stimuli at a population level. We hypothesized that activity patterns of population SC neurons could better discriminate the face-like patterns than activity of individual SC neurons (see below).

One of the long-held views of SC’s role in visual detection is that it is not involved in form detection such as faces. The results of the present study challenge this view, since they indicated that the SC neurons showed preferential responses to the face-like patterns; SC neurons responded stronger and faster to the face-like than non-face patterns in both the white and black stimulus sets regardless of face orientation (upright or inverted). Furthermore, response magnitudes and latencies to the white stimulus set were correlated with those to the black stimulus set. The MDS results further indicated that face-like patterns were significant, if not completely, separated from other stimuli within 50 ms after stimulus onset and that upright and inverted face-like patterns were separated from the other stimuli regardless of contrast polarity of the stimuli and regardless of face orientation in epoch 2. These results indicated that SC neurons responded in a uniform manner and stronger to the white and black face-like patterns in short latencies. Furthermore, it suggests that the SC neurons might be involved in the detection of face-like patterns regardless of contrast polarity of the stimuli and regardless of face orientation before the cortical face processing system operates. Some findings of recent studies are consistent with the present results. A behavioral study reported the existence of a broadly tuned face-detection system in monkeys consistent with human studies (Taubert et al., 2017). A neurophysiological study reported that rat SC neurons responded similarly to normal and contrast-reversed circles, suggesting that the SC might detect edges of forms (Girman and Lund, 2007), which is important for object detection and form processing (Kubilius et al., 2014). Furthermore, a neuropsychological study using a human blindsight patient due to removal of the cerebral cortex, pulvinar, and lateral geniculate body but not the SC in the affected hemisphere reported that the SC represented configuration of multiple stimuli and responded differentially to gestalt-like complex assemblies of stimuli (Georgy et al., 2016). Consistent with this finding, configural processing of face features is required for face detection in the initial stage of face information processing (see “Introduction” section). Taken together, the results strongly suggest that the SC neurons are sensitive to first-order information of faces required for face detection.

The SC neurons responded similarly to the upright and inverted face-like patterns, suggesting that preferential responses to the face-like pattern were not ascribed to differences in the spatial location of particular edges. This finding is consistent with human neuropsychological study reporting similar sensitivity to both inverted and upright faces in the subcortical pathway (Sato et al., 2012; Gabay et al., 2014), while cortical face-related areas such as the fusiform cortex are sensitive to vertical inversion (Mazard et al., 2006; Nasr and Tootell, 2012). Furthermore, the effects of vertical inversion (a decrease in face recognition due to vertical inversion) become more evident with age in human children (de Heering et al., 2012). These findings suggest that sensitivity to upright faces in the cortical face areas gradually increases due to daily exposure to facial stimuli, while the subcortical visual pathway might be less prone to plastic changes. On the other hand, the face-like patterns in the black stimulus set are the contrast-reversed stimuli of those in the white stimulus set. Although contrast polarity is reported to be important for recognition of face identity rather than face itself (Harris et al., 2014), contrast reversal significantly affects face detection; a human neurophysiological and fMRI studies suggest that contrast reversal increased detection threshold and decreased signal-to-noise ratio in the cortical face processing system (Nasr and Tootell, 2012; Liu-Shuang et al., 2015). These findings are consistent with the present results, in which ratios of the response magnitudes to the face-like patterns to those to the blanks (selectivity to face-like patterns) were larger in the white stimulus set (positive face-like patterns) than the black stimulus set (negative face-like patterns) in the SC neurons with the central RFs, and also consistent with a behavioral study in which newborn babies showed preference to positive face-like patterns compared with negative face-like patterns (Farroni et al., 2005). These results suggest that sensitivity to positive face-like patterns in the subcortical visual system might explain partially superior responses to positive faces in adults since the subcortical visual system affects neurophysiological responses to positive and negative faces in the cortical face areas in adult humans (Tomalski and Johnson, 2012).

### Effects of RFs on Face Information Processing

Although all SC neurons with different response areas (upper, lower or central RFs) responded stronger to the face-like patterns than the non-face patterns, SC neurons with the central RFs showed responses more selective to the face-like patterns; (1) relative response magnitudes to the face-like patterns were larger in the SC neurons with the central RFs than those with the peripheral RFs; and (2) more SC neurons with the central RFs showed significant correlation between responses to the white and black stimulus sets than those with the peripheral RFs. Furthermore, the MDS analysis of the SC neurons with the central RFs also indicated that the face-like patterns were separated from the remaining stimuli in epochs 1 and 2. These results suggest that faces are differently represented in the SC depending on eccentric stimulus locations. Consistently, recent data suggest that visual object recognition is dependent on its retinal position (Kravitz et al., 2008), and faces are represented in the foveal area in the occipito-temporal cortices (e.g., fusiform face area; Levy et al., 2001). A recent neurophysiological study reported that monkey SC neurons are highly sensitive to stimuli in the foveal VF than previously reported (Chen et al., 2019). Furthermore, fMRI studies reported a similar central bias for faces in the human SC as well as the amygdala in contrast with non-face objects (Almeida et al., 2013, 2015). The present results, as well as the previous imaging studies in the human SC and amygdala (Almeida et al., 2013, 2015), suggest that faces are also associated with central-biased representation in the subcortical visual system.

Furthermore, mean response latencies of the SC neurons with the peripheral RFs were shorter than those of the SC neurons with the central RFs, and those of the SC neurons with the upper RFs were shorter than those of the SC neurons with the central and lower RFs. A previous neurophysiological study also reported faster and stronger responses in monkey SC neurons with upper RF (Hafed and Chen, 2016). These results might be associated with developmental and behavioral backgrounds; the upper VF seems to be more sensitive to visual objects in newborn babies (Simion et al., 2002), and faces are efficiently recognized in the upper VF (Sheperd et al., 1981). Furthermore, the upper VF, compared with the lower VF, has been suggested to be associated with visual search and object recognition (Previc, 1990), and saccadic latencies were shorter when static targets were presented in the upper VF (Heywood and Churcher, 1980). These findings suggest that the SC might be important to orient to faces in the upper VF.

Finally, we found a topographical distribution of SC neurons; neurons with the upper RF were located mainly at the medial parts of the SC, neurons with lower RFs, at the lateral parts of the SC, and neurons with the central RF, at the anterior-lateral parts of the SC. This distribution pattern of the SC neurons is consistent with the previous studies (Cynader and Berman, 1972; Goldberg and Wurtz, 1972). It has been suggested that the SC plays an important role in mapping stimulus saliency in the space (Horwitz and Newsome, 1999; McPeek and Keller, 2004; Krauzlis et al., 2013; Veale et al., 2017; White et al., 2017), and luminance, motion, and color of the stimulus are critical stimulus features for stimulus saliency in patients with V1 lesions (Itti and Koch, 2001; Yoshida et al., 2012). The present results suggest that the SC contributes to the formation of an innate biological saliency map that incorporates not only stimulus physical features but also crude form information including faces, which enables newborn babies and newly hatched and dark-reared chicks to orient to face-like patterns. Bilateral lesions of the SC decreased social responses to conspecifics (Maior et al., 2012), suggesting the importance of this structure in facial recognition during early life. A study in a patient with unilateral lesion of the amygdala, the downstream structure of the subcortical pathway, showed deficits in reflective saccade toward facial stimuli during brief stimulus presentations (Gamer et al., 2013), suggesting the importance of the visual inputs from the SC to the amygdala in orienting toward facial stimuli. Taken together, the present results provide neurophysiological evidence for the SC involvement in innate recognition of facial stimuli.

## Conclusion

The present neurophysiological results revealed that preferential responses of monkey SC neurons to face-like patterns. This finding is consistent with phylogenetic and ontogenetic evidence for the SC involvement in innate face detection. Thus, the present evidence provides a neurophysiological basis for a suggestion that a subcortical facial processing system in vertebrates has been proposed to function as a prototypical face template, i.e., “Conspec” (Morton and Johnson, 1991). Consistently, the SC neurons responded best to the face-like patterns in spite of four different contours in the present study.

Previous neurophysiological studies reported that response magnitudes and latencies of monkey pulvinar neurons to various visual stimuli including facial photos were correlated to those of the monkey SC neurons (Nguyen et al., 2013, 2014). These findings suggest that the SC and pulvinar constitute the subcortical visual pathway for innate face detection. Neuroanatomical, non-invasive imaging and neurophysiological studies reported existence of this subcortical pathway consisting of the SC, pulvinar, and amygdala in animals and humans (Linke et al., 1999; Day-Brown et al., 2010; Tamietto et al., 2012; Garvert et al., 2014; Rafal et al., 2015; Elorette et al., 2018; Kinoshita et al., 2019). These findings suggest that the SC is a first node in the subcortical pathway, where retinal inputs are integrated into facial information.

## Data Availability Statement

The datasets generated for this study are available on request to the corresponding author.

## Ethics Statement

The animal study was reviewed and approved by the Committee for Animal Experiments and Ethics at the University of Toyama.

## Author Contributions

HNishij conceived the study and designed the experiment. QuangL and QuanL performed the experiment. QuangL, QuanL, and HNishij analyzed data and wrote the article. HNishij, HNishim, JM, YT, EH, RM, CT, and TO revised the article. All the authors discussed the results and commented on the manuscript, and read and approved the final manuscript.

## Conflict of Interest

The authors declare that the research was conducted in the absence of any commercial or financial relationships that could be construed as a potential conflict of interest.
