# Supplementary material for: A Prototypical Template for Rapid Face Detection Is Embedded in the Monkey Superior Colliculus
Source: Front Syst Neurosci. 2020 Feb 6;14:5. doi: 10.3389/fnsys.2020.00005 (PMC7025518; doi:10.3389/fnsys.2020.00005)
Supplement: Supplementary file 1 [file Data_Sheet_1.PDF]

Supplementary Materials

Supplementary Results

Coherent responses to face-like patterns across different SC layers and different RFs

We confirmed that similar coherent responses to the white and black face-like patterns were replicated when the data in different SC layers were separately analyzed. The responsive SC neurons tested with all the stimuli were divided into two groups based on their locations: those in the superficial (n = 80) and deep (n = 66) layers. Supplementary Figure 4 shows response magnitudes of neurons in the superficial (A) and deep (B) layers to the white (a) and black (b) stimulus sets, respectively. The mean response magnitudes to the face-like patterns were significantly larger than those to the non-face patterns in both the superficial (A) and deep (B) layers in both the white (a) and black (b) stimulus sets (Bonferroni tests after repeated measures two-way ANOVA; $P < 0.001$, $P < 0.05$ for the white and black stimulus sets in the superficial layer, respectively; $P < 0.01$, $P < 0.05$ for the white and black stimulus sets in the deep layer, respectively). Furthermore, statistical analysis by simple linear regression indicated that there were significant positive correlations between the mean response magnitudes to the white and black stimulus sets in the superficial [$F(1, 18) = 85.451$, $P < 0.0001$; $r = 0.909$] (Ac) and deeper [$F(1, 18) = 103.473$, $P < 0.0001$; $r = 0.923$] (Bc) layers. The results indicated that response characteristics of the SC neurons in all the examined layers were dependent on the stimulus form although stimulus selectivity was low.

Similar coherent responses to the white and black face-like patterns were also confirmed when the neurons with different RFs were separately analyzed. Supplementary Figure 5 shows response magnitudes to the white (A) and black (B) stimulus sets in the SC neurons with lower (n = 68) (a), upper (n = 46) (b), and central (n = 32) (c) RFs. The results showed similar results: the mean response magnitudes of the SC neurons with three different RFs to the face-like patterns were significantly larger than those to the nonface patterns in both white (A) and black (B) stimulus sets (Bonferroni tests after repeated measures two-way ANOVA; $P < 0.01$, 0.001, 0.01 for lower (a), upper (b), and central (c) RFs, respectively, in the white stimulus set; $P < 0.001$, 0.05, 0.05 for lower (a), upper (b), and central (c) RFs, respectively, in the black stimulus set). Supplementary Figure 6 shows the relationships between response magnitudes to the white and black stimuli of the same forms. Statistical analyses by simple linear regression indicated that there were significant positive correlations between the mean response magnitudes to the white and black stimulus sets in the SC neurons with lower RFs [$F(1, 18) = 115.766$, $P < 0.0001$; $r = 0.930$] (A); upper RFs [$F(1, 18) = 24.410$, $P < 0.0001$; $r = 0.759$] (B); and central RFs [$F(1, 18) =$

255.451, $P < 0.0001$; $r = 0.967$] (C). Supplementary Figure 7 shows response latencies to the white (A) and black (B) stimulus sets in the SC neurons with the lower (n = 54) (a), upper (n = 39) (b), and central (n = 22) (c) RFs. The results showed similar results: the mean response latencies of the SC neurons with three different response areas to the face-like patterns were significantly shorter than those to the nonface patterns in both white (A) and black (B) stimulus sets (Bonferroni tests after repeated measures two-way ANOVA; $P < 0.05$, 0.01, 0.05 for lower (a), upper (b), and central (c) RFs, respectively, in the white stimulus set; $P < 0.01$, 0.05, 0.05 for lower (a), upper (b), and central (c) RFs, respectively, in the black stimulus set). Supplementary Figure 8 shows the relationships between response latencies to the white and black stimuli of the same forms. Statistical analyses by simple linear regression indicated that there were significant positive correlations between the mean response latencies to the white and black stimulus sets in the SC neurons with lower RFs [$F(1, 18) = 13.189$, $P = 0.004$; $r = 0.650$] (A), upper RFs [$F(1, 18) = 12.611$, $P = 0.005$; $r = 0.642$] (B), and central RFs [$F(1, 18) = 24.968$, $P < 0.0001$; $r = 0.762$] (C). These analyses indicated essentially similar results, in which the SC neurons (regardless of their RFs) responded stronger and faster to the face-like patterns than the non-face patterns (regardless of contrast polarity).

However, we found significant differences in the mean response latencies of SC neurons between SC neurons with different RFs and SC neurons in different layers (Supplementary Figure 9). When responses latencies of the SC neurons in the short latency group (n = 143) were analyzed by a two-way ANOVA with "contrast polarity" (white vs. black) and "RFs" [central (n = 32) vs. peripheral (n = 111)] as factors, the mean response latencies to the white and black stimulus sets were significantly longer in the SC neurons with the central RFs than those with the peripheral RFs [significant main effect of RFs in a two-way ANOVA; $F(1, 282) = 84.573$, $P < 0.0001$] (A). Furthermore, when response latencies were analyzed by a two-way ANOVA with "contrast polarity" and "RFs" [lower (n = 68) vs. upper (n = 43)] as factors, the mean response latencies to the white and black stimulus sets were significantly longer in the SC neurons with lower RFs than those with the upper RFs [significant main effect of RFs in a two-way ANOVA; $F(1, 218) = 121.428$, $P < 0.0001$] (B). When the response latencies were analyzed by a two-way ANOVA with "contrast polarity" and "SC layer" [deeper (n = 65) vs. superficial (n = 78)] as factors, the mean response latencies to the white and black stimulus sets were significantly longer in the SC neurons in the deeper layers than those in the superficial layers [significant main effect of RFs in a two-way ANOVA; $F(1, 282) = 8.318$, $P = 0.004$] (C).

Supplementary figures

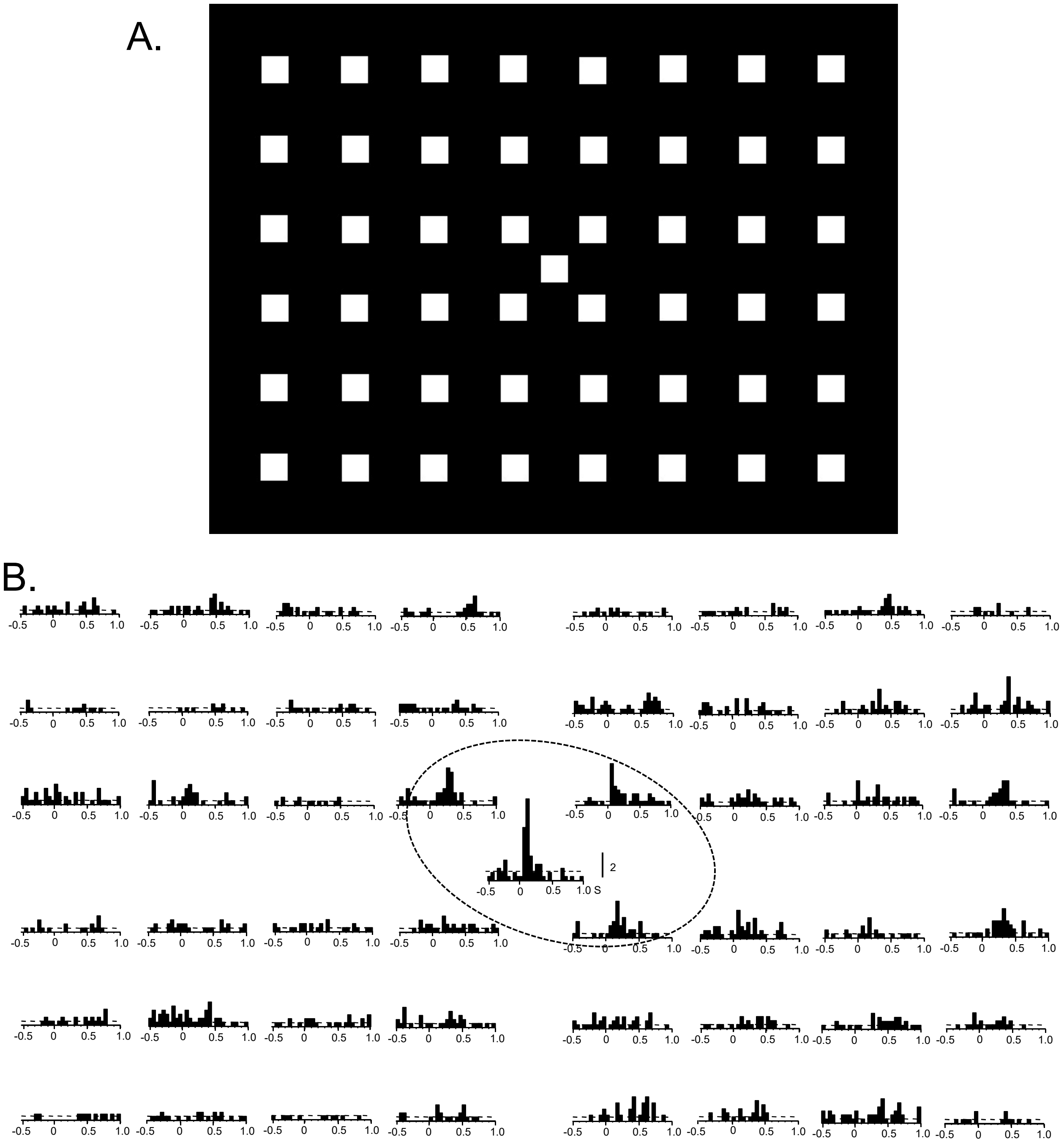

Supplementary Figure 1. Arrangement of the visual stimuli to identify the largest response fields of SC neurons (A) and an example of an SC neuron with the largest response field in the center of the display (B).

A: A white square was randomly presented for 500 ms at different locations on the display to identify the largest response field. B: Each histogram represents a perievent summed histogram of an SC neuron showing responses to the white square at each location on the display. Three histograms within a dotted circle show significant responses with the largest response field at the center of the display. Calibration at the center of the figure: number of spikes per trial in each bin. Bin width, 50 ms. Zero on the abscissa indicates onset of the stimuli. Each dotted line in each histogram indicates a mean spontaneous firing rate.

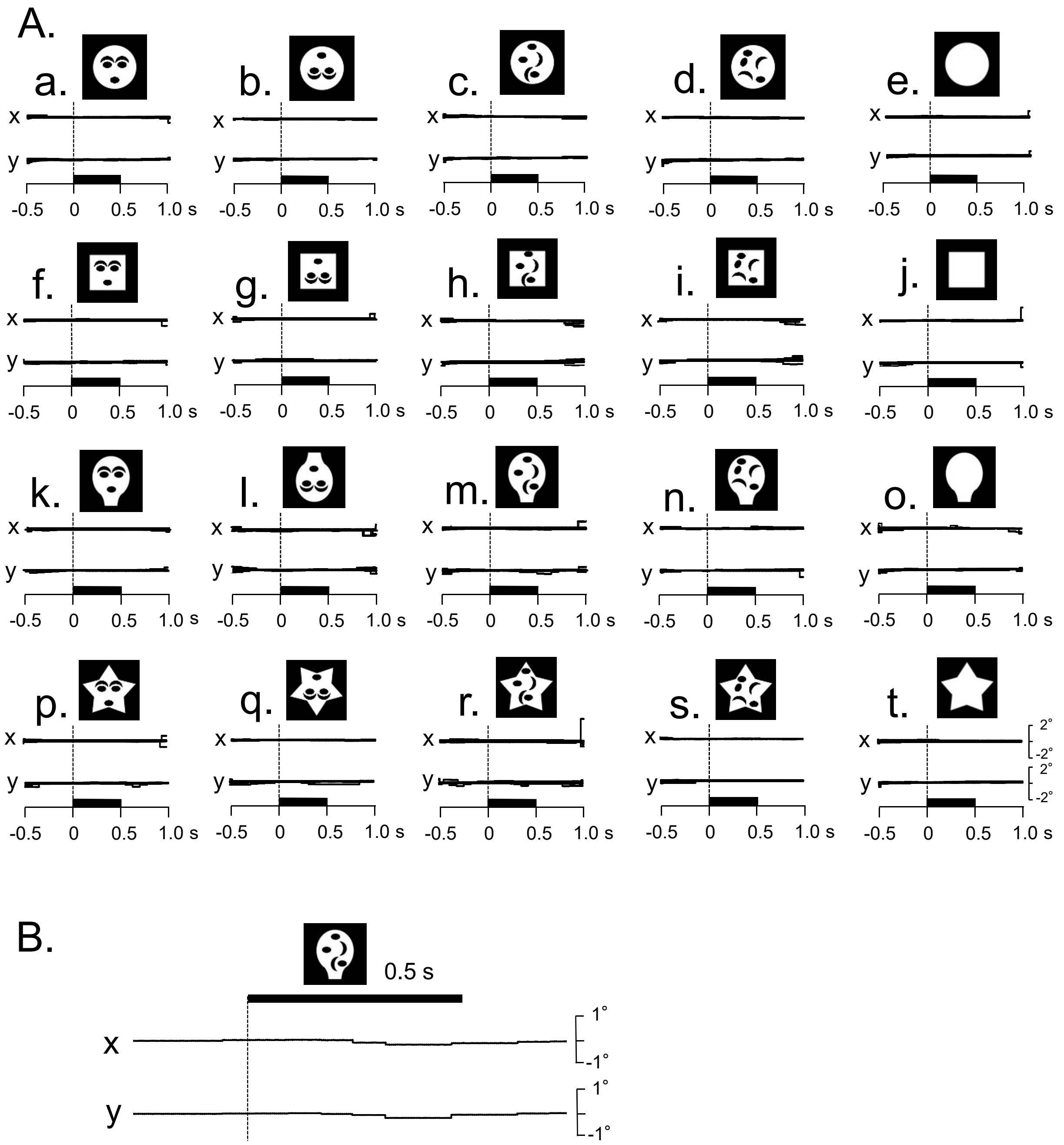

Supplementary Figure 2. Examples of eye position traces.

Aa-t: Superimposed (5-12 trials in each stimulus) traces of eye positions during recording of an SC neuron in response to the white stimuli. Thick lines on the time scales indicate the stimulus presentation period. B: Traces of eye positions in one trial in Am. Three microsaccades are observed during stimulus presentation in this trial.

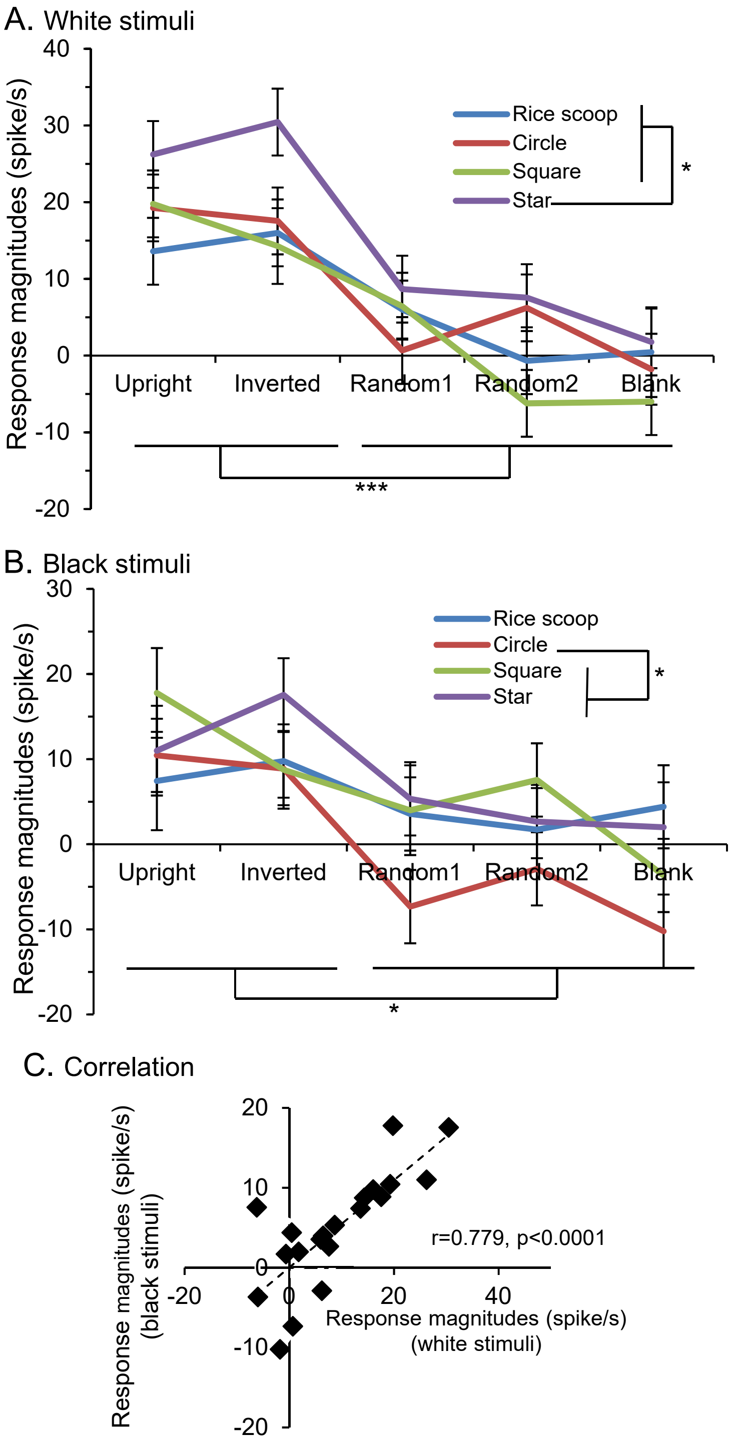

Supplementary Figure 3. Comparison of the response magnitudes of the same neuron shown in Figure 2.

A: Comparison of the response magnitudes to the white stimulus set among the five visual stimuli. *, ***, significant difference by Bonferroni tests after a two-way ANOVA at $p < 0.05$, and $p < 0.001$, respectively. B: Comparison of the response magnitudes to the black stimulus set among the five visual stimuli. *, significant difference by Bonferroni tests after a two-way ANOVA at $p < 0.05$. C: Significant correlation of response magnitudes between the white and black stimulus sets [$F(1, 18) = 27.843$, $p < 0.0001$; $r = 0.779$].

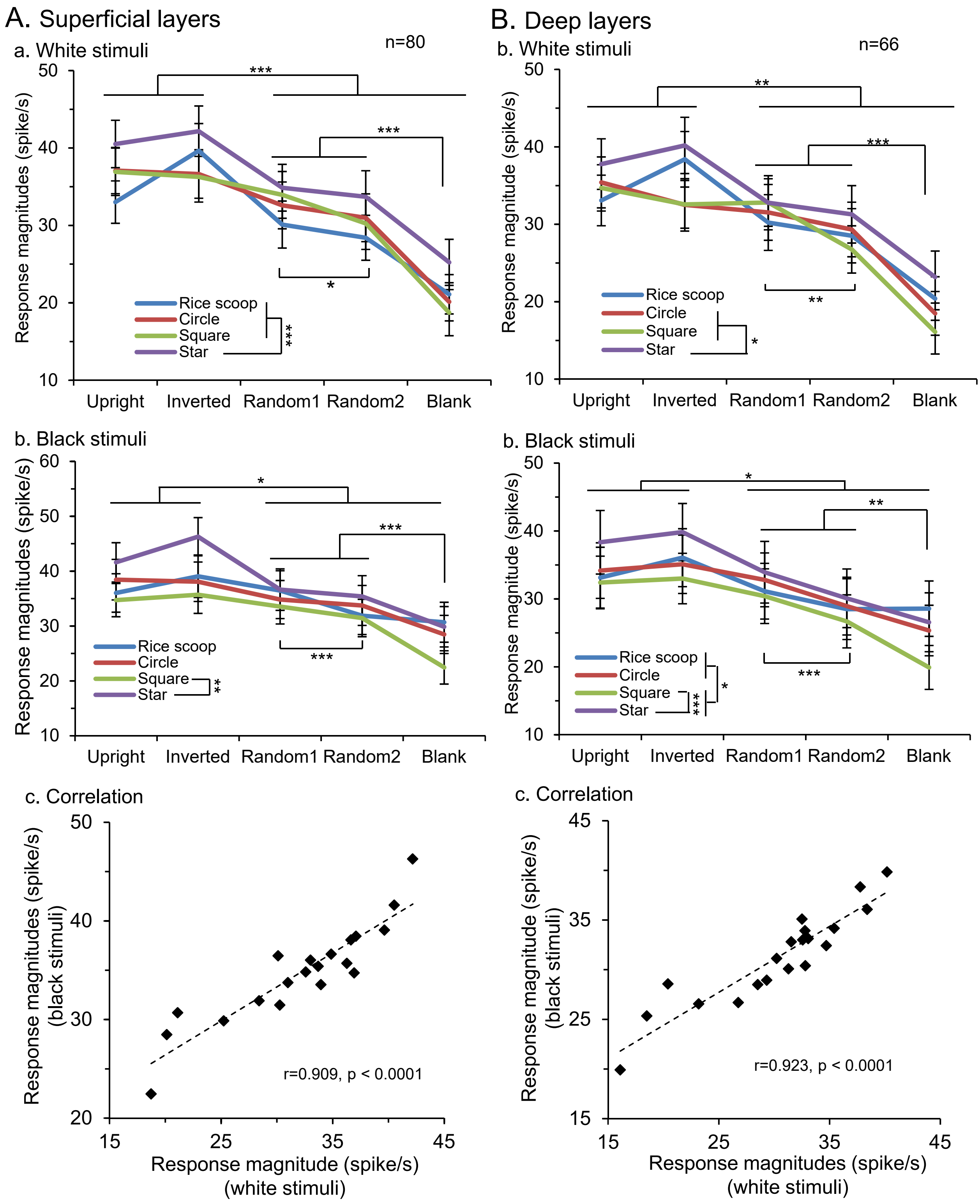

Supplementary Figure 4. Comparison of the response magnitudes of the SC neurons in the superficial (A) and deep (B) layers among the five visual stimuli.

A, B: Comparison of the response magnitudes to the white (a) and black (b) stimulus sets among the five visual stimuli, and correlation of the response magnitudes between the white and black stimulus sets (c). *, **, ***, significant difference by Bonferroni tests after a repeated measures two-way ANOVA at $p < 0.05$, $p < 0.01$, and $p < 0.001$, respectively.

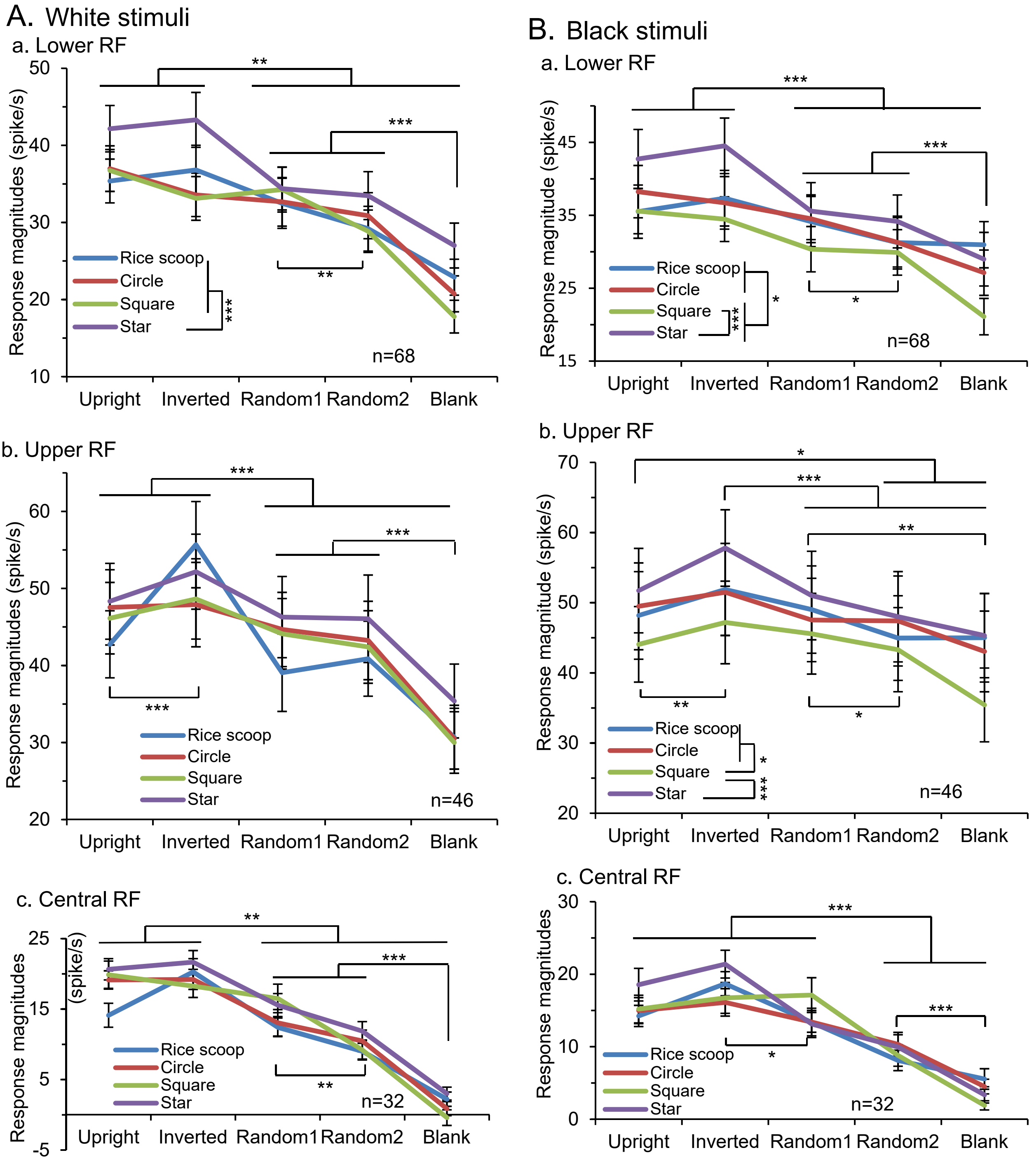

Supplementary Figure 5. Comparison of the response magnitudes of the SC neurons with different RFs to the white (A) and black (B) stimulus sets among the five visual stimuli.

A, B: Comparison of the response magnitudes of the SC neurons with the lower (a), upper (b), and central (c) RFs among the five visual stimuli. *, **, ***, significant difference by Bonferroni tests after a repeated measures two-way ANOVA at $p < 0.05$, $p < 0.01$, and $p < 0.001$, respectively.

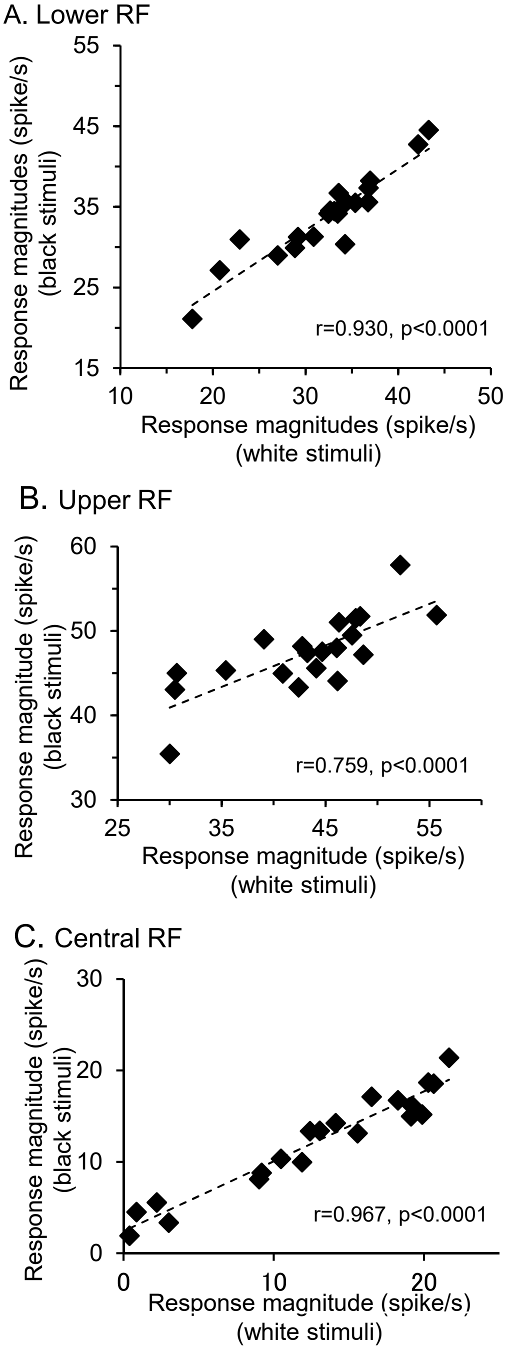

Supplementary Figure 6. Correlations of the response magnitudes between the white and black stimulus sets in the SC neurons with the lower (A), upper (B), and central (C) RFs.

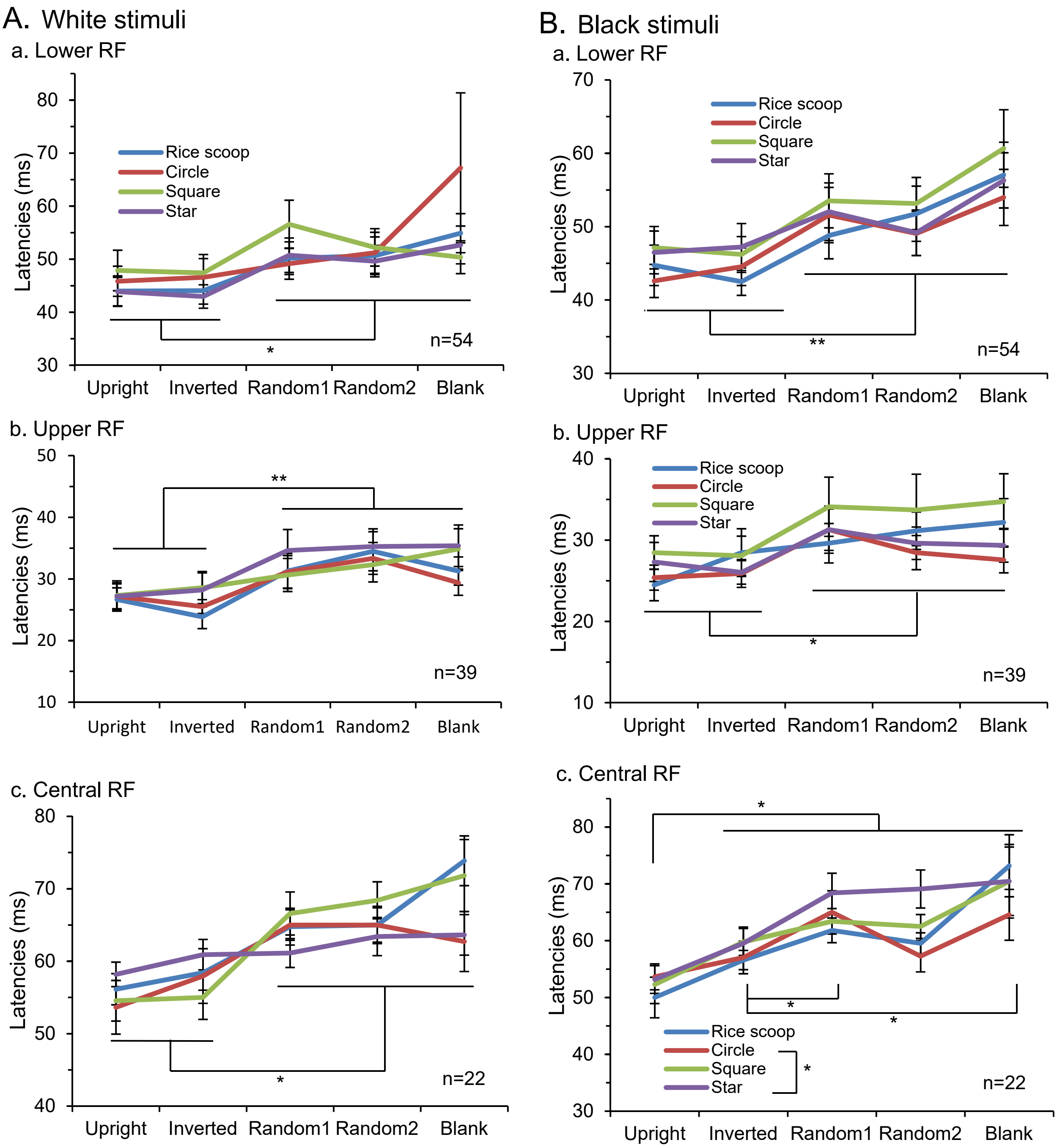

Supplementary Figure 7. Comparison of the response latencies of the SC neurons with different RFs among the five visual stimuli in the white (A) and black (B) stimulus sets.

A, B: Comparison of the response latencies of the SC neurons with the lower (a), upper (b), and central (c) RFs among the five visual stimuli. *, **, significant difference by Bonferroni tests after a repeated measures two-way ANOVA at $p < 0.05$, and $p < 0.01$, respectively.

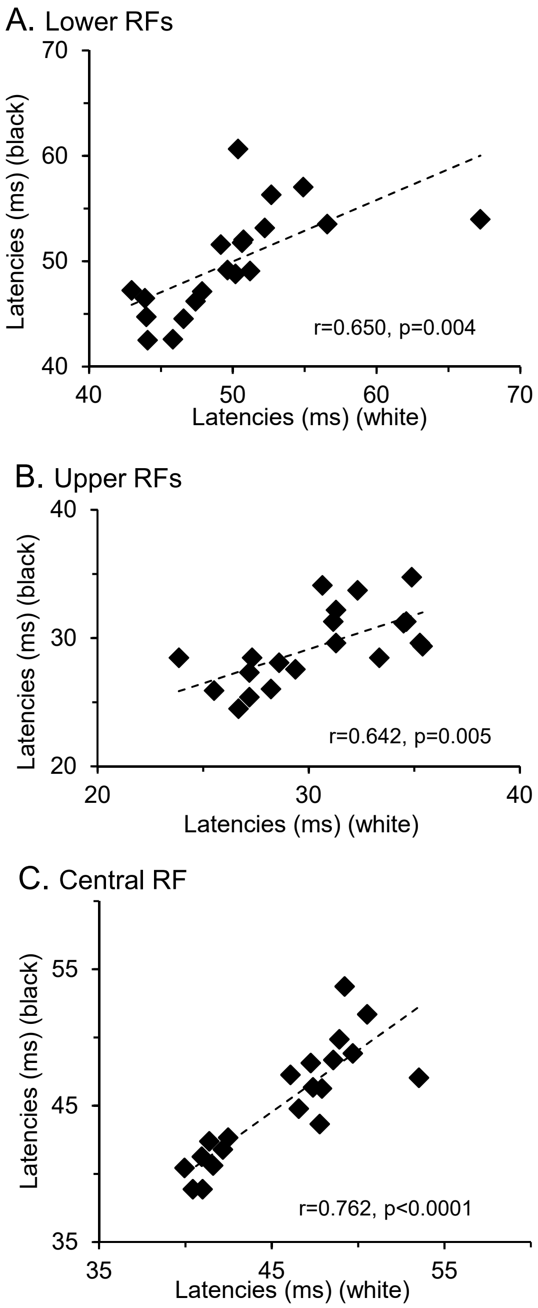

Supplementary Figure 8. Correlations of the response latencies between the white and black stimulus sets in the SC neurons with the lower (A), upper (B), and central (C) RFs.

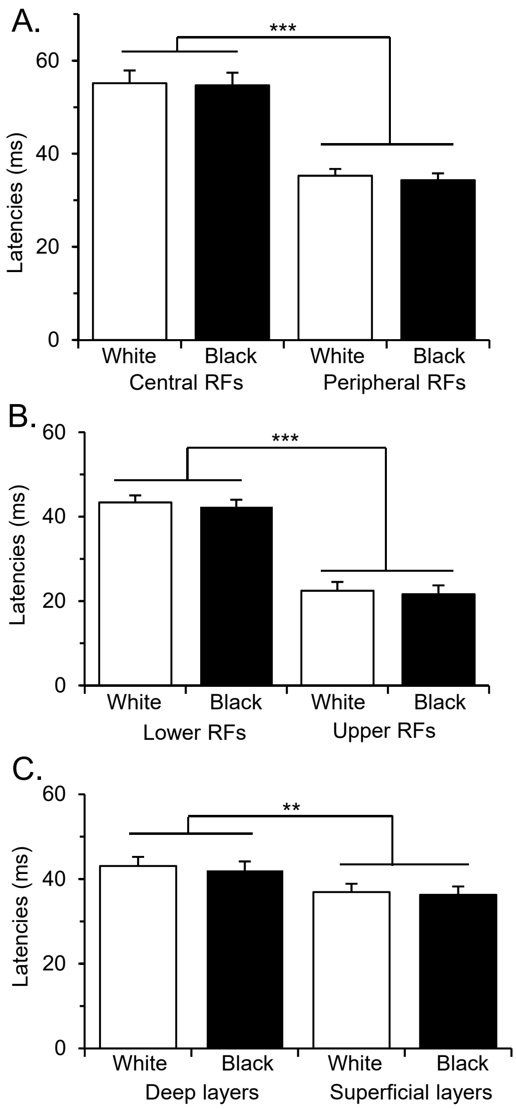

Supplementary Figure 9. Comparison of response latencies between the SC neurons with the central RFs and those with the peripheral RFs (A), between those with the lower and upper RFs (B), and between the SC neurons in the superficial and deep layers (C).

White, white stimuli; black, black stimuli. **, ***, significant main effect in a two-way ANOVA at $p < 0.01$, and $p < 0.001$, respectively.
